# Role of Th22 Cells in the Pathogenesis of Autoimmune Diseases

**DOI:** 10.3389/fimmu.2021.688066

**Published:** 2021-07-06

**Authors:** Qi Jiang, Guocan Yang, Fan Xiao, Jue Xie, Shengjun Wang, Liwei Lu, Dawei Cui

**Affiliations:** ^1^ Department of Blood Transfusion, Shaoxing People’s Hospital (Shaoxing Hospital, Zhejiang University School of Medicine), Shaoxing, China; ^2^ Department of Pathology and Shenzhen Institute of Research and Innovation, The University of Hong Kong; Chongqing International Institute for Immunology, Chongqing, China; ^3^ Department of Blood Transfusion, The First Affiliated Hospital, Zhejiang University School of Medicine, Hangzhou, China; ^4^ Department of Laboratory Medicine, The Affiliated People’s Hospital, Jiangsu University, Zhenjiang, China; ^5^ Department of Immunology, Jiangsu Key Laboratory of Laboratory Medicine, School of Medicine, Jiangsu University, Zhenjiang, China

**Keywords:** Th22 cells, IL-22, rheumatoid arthritis, systemic lupus erythematosus, psoriasis, immune thrombocytopenia, autoimmune diseases, autoimmune hepatitis

## Abstract

Upon antigenic stimulation, naïve CD4^+^T cells differentiate into different subsets and secrete various cytokines to exert biological effects. Th22 cells, a newly identified CD4^+^T cell subset,are distinct from the Th1, Th2 and Th17 subsets. Th22 cells secrete certain cytokines such as IL-22, IL-13 and TNF-α, but not others, such as IL-17, IL-4, or interferon-γ (IFN-γ), and they express chemokine receptors CCR4, CCR6 and CCR10. Th22 cells were initially found to play a role in skin inflammatory diseases, but recent studies have demonstrated their involvement in the development of various autoimmune diseases. Here, we review research advances in the origin, characteristics and effector mechanisms of Th22 cells, with an emphasis on the role of Th22 cells and their main effector cytokine IL-22 in the pathogenesis of autoimmune diseases. The findings presented here may facilitate the development of new therapeutic strategies for targeting these diseases.

## Introduction

CD4^+^ T cells mainly include Th1, Th2, Th17, Th9, Th22, follicular helper T (Tfh) cells and regulatory T (Treg) cells (1–4), and they were initially divided into two categories: Th1 and Th2 cells (5). This classification does not explain the pathogenesis of certain diseases; for example,neutralizing or eliminating IL-12 or Th1 cells and the cytokine IFN-γ could not prevent or alleviate experimental autoimmune encephalomyelitis (EAE) and collagen-induced arthritis (CIA); however, the discovery of Th17 cells explains this paradox. Th17 cells do not express IL-4 or IFN-γ but secrete IL-17 at a high level (6, 7). Previous studies have shown that both Th17 and Th1 cells secrete IL-22 (8, 9). A recent report identified that a group of special T cell subsets that could secrete IL-22 and IL-13 but not IL-17 and IFN-γ (10). Mouse T lymphoma cells stimulated with IL-9 expressed a cytokine very similar to the secondary structure of IL-10, and it is named interleukin-10-related T cell-derived inducible factor (IL-TIF) (11). Further studies identified a new sequence from human T cells that encodes 23% amino acids homologous to IL-10 and is 87% similar to IL-TIF, and it is named IL-22 (12). The expression of IL-22 has been suggested to be associated with Th1 (8, 13) and Th17 cells (9, 14). However, Th22 cell clones in the induced environment of Th1, Th2, Th17 and Treg cells do not secrete their characteristic cytokines but can secrete IL-22, which demonstrates that Th22 cells represent an independent T cell subset. It is named Th22 because of its high level of IL-22 secretion (15). Plank et al. performed a whole gene chip analysis and found differences between Th22 and Th17 cells, and they determined that Th22 cells were an independent cell lineage (16).

Th22 cells can differentiate into Th1 or Th2 cells under appropriate conditions. In an *in vivo* IFN-γ-rich inflammatory microenvironment or *in vitro* Th1-promoting conditions, Th22 cells have obvious plasticity. In an *in vitro* Th2 culture environment, Th22 cells showed increased secretion of IL-13 (16). The conditions for the transformation between Th22 and Th17 cells have not been clarified (17). Since other cells, such as Th17 cells, also secrete IL-22, the identification of Th22 cells is particularly important. Mousset et al. suggested that when using flow cytometry to identify Th22 cells, Th22 cells can be identified by integrating cell surface markers (CCR4^+^, CCR6^+^ and CCR10^+^), combining cell transcription factors (AhR^+^ and/or STAT3^+^) and/or cytokine staining (IL-22^+^), and requiring IFNγ^-^, IL- 4^-^, IL-9^-^, IL-10^-^ and IL-17^-^ (18). Here, we review the characteristics and effector mechanisms of Th22 cells, with an emphasis on the role of Th22 cells and their main effector cytokine IL-22 in the pathogenesis of autoimmune diseases, including rheumatoid arthritis (RA), systemic lupus erythematosus (SLE), psoriasis,multiple sclerosis (MS), immune thrombocytopenia (ITP), immunoglobulin A nephropathy (IgAN), autoimmune hepatitis (AIH), autoimmune thyroid diseases (AITD), myasthenia gravis (MG), and systemic sclerosis (SSc).

## Characteristics of Th22 Cells

Th22 cells secrete IL-22, IL-13, IL-26, TNF-α and granzyme B but not IL-17, IFN-γ or IL-4 (16). Activation of the transcription factor aryl hydrocarbon receptor (AhR) significantly promotes the differentiation of naïve CD4^+^T cells into Th22 cells (19). The expression of the signature cytokines and transcription factors of Th1, Th2 and Th17 cells was absent in Th22 cells (10, 15, 19). Th22 cells are abundant in human skin and play important roles in epidermal wound healing (15). These cells are tissue homing CD4^+^T cells and exhibit anti-inflammatory, antibacterial and antiviral activities. Emerging evidence has shown the critical roles of Th22 cells in allergies, autoimmune diseases, intestinal diseases and tumors (20–22).

IL-22 is the main effector molecule of Th22 cells and belongs to the IL-10 family, and it acts by binding to IL-22 receptors which are composed of IL-22R1 (main high affinity chain) and IL-10R2 (helper receptor chain) subunits (23). IL-22R1 is mainly expressed in nonhematopoietic organs, such as the skin, lung,intestine and pancreas, but not in lymphoid organs including thymus, bone marrow and spleen (24). IL-22 has little effects on immune cells, and mainly acts on mucosal barriers of skin,respiratory system and digestive system. IL-22 binding protein (IL-22BP) or IL-22RA2, the soluble receptor of IL-22, is expressed in various tissues around the lung, colon and breast. IL-22BP blocks the binding of IL-22 to IL-22R and is the receptor antagonist of IL-22 (25). IL-22 can also be produced by lymphoid cells, including Th17 cells, innate lymphocytes (ILCs), dermis γδ T cells, Tc17 cells, nonlymphatic macrophages (26), neutrophils (27–29) and even fibroblasts of RA patients (28, 30–33). IL-22 in the intestine is mainly produced by ILCs. ILCs migrate from mucosal associated lymphoid tissue to the lamina propria after stimulation (12). γδT cells are the primary source of IL-22 in the skin, intestinal tract, lung, reproductive tract and other epithelial tissues, where they respond quickly to exotic pathogens at an early stage (34, 35). Moreover, IL-22 can induce the production of different antimicrobial proteins by keratinocytes, intestinal epithelial cells,bronchial epithelial cells and other different parts of the human body (36). In addition, IL-22 can directly act on endothelial cells through the activation of STAT3 and ERK pathways,stimulate endothelial cell proliferation and migration,and stimulate angiogenesis (37). IL-22 can also act on colonic subepithelial myofibroblasts (SEMFs) to produce inflammatory mediators such as chemokines, inflammatory cytokines and matrix metalloproteinases (MMPs) (38).

**Figure 1:**
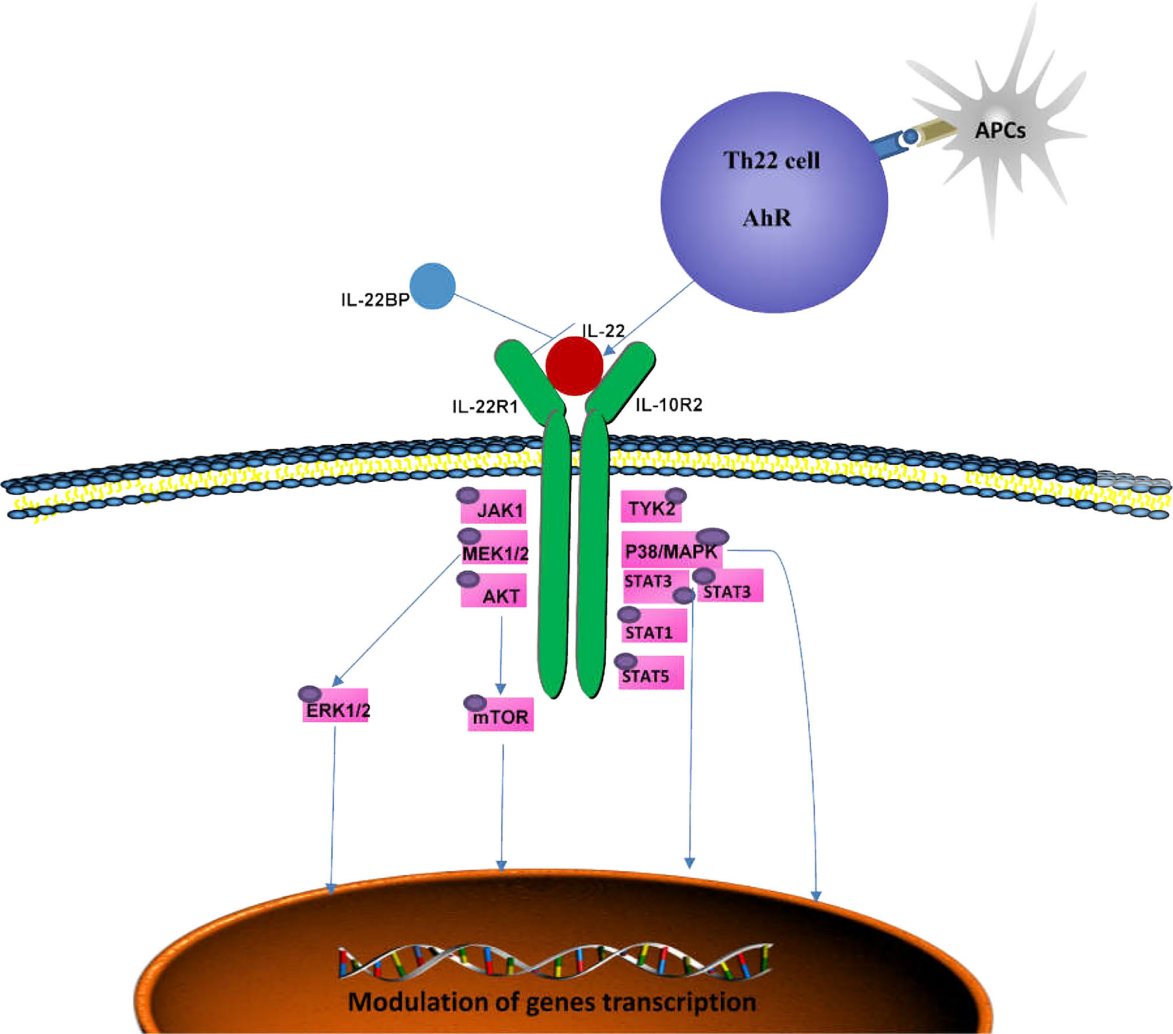
Signaling pathways mediated by IL-22. The IL-22 receptor complex consists of IL-22R1 and IL-10R2. By binding to its receptor, IL-22 activates TYK2 and JAK1and triggers multiple intracellular pathways, including p38MAPK, MEK1/2, ERK1/2 and AKT, by phosphorylating serine and tyrosine in STAT3, STAT1 and STAT5, which can ultimately lead to immune homeostasis. IL-22BP is a soluble receptor antagonist that specifically neutralizes the activity of IL-22. AhR, aryl hydrocarbon receptor; APCs, antigen-presenting cells; JAK1, Jenus kinase 1; TYK2, tyrosine kinase 2; MAPK, mitogen-activated protein kinase; IL-22BP, IL-22 binding protein.

The binding of IL-22 to IL-22R activates downstream signal transduction (**Figure 1**) (39). IL-22 transmits phosphorylation signals downstream through Janus kinase (JAK) 1 and tyrosine kinase (TYK) 2, including the mitogen-activated protein kinase (MAPK) pathway (p38 kinase, ERK1/2, MEK1/2 and JNK),STAT3, STAT1 and STAT5 (39–41). Similar to other members of the IL-10 family, IL-22 phosphorylates STAT3 mainly at Ser727 and Tyr705 (23, 42, 43). IL-22 is also unique in that it induces the phosphorylation of serine residues in addition to tyrosine residues, and activates the ERK1/2 pathway (40), whereas IL-10 induces the phosphorylation of tyrosine residues on STAT3. This difference may be caused by the difference inreceptor R1. The binding of SRC homologous phosphatase 2 (Shp2) to Tyr-251 phosphorylation residues and the activation of IL-22R1 on Tyr-301 are necessary for the activation of STAT3 (39, 44). Moreover, the phosphorylation of STAT3 is essential for IL-22 to exert its effects on epithelial cells (45). IL-22 also induces the expression of suppressor cytokine signaling1 and 3 (SOCS1/3), which in turn inhibits the activity of STAT3 (39). Activation of STAT1 and/or STAT5 can be observed in tumors (46–48). IL-22 can also activate the PI3K-Akt-mTOR pathway, which is essential for the migration of hepatocytes and colonic epithelial cells (39, 49). The activation of Akt is important for the proliferation of human fibroblast-like synoviocytes and epidermal keratinocytes (50). IL-22 induces osteoclast formation in RA by p38MAPK/NF-κB and JAK2/STAT3 signaling (51).

## Differentiation of Th22 Cells

**Figure 2:**
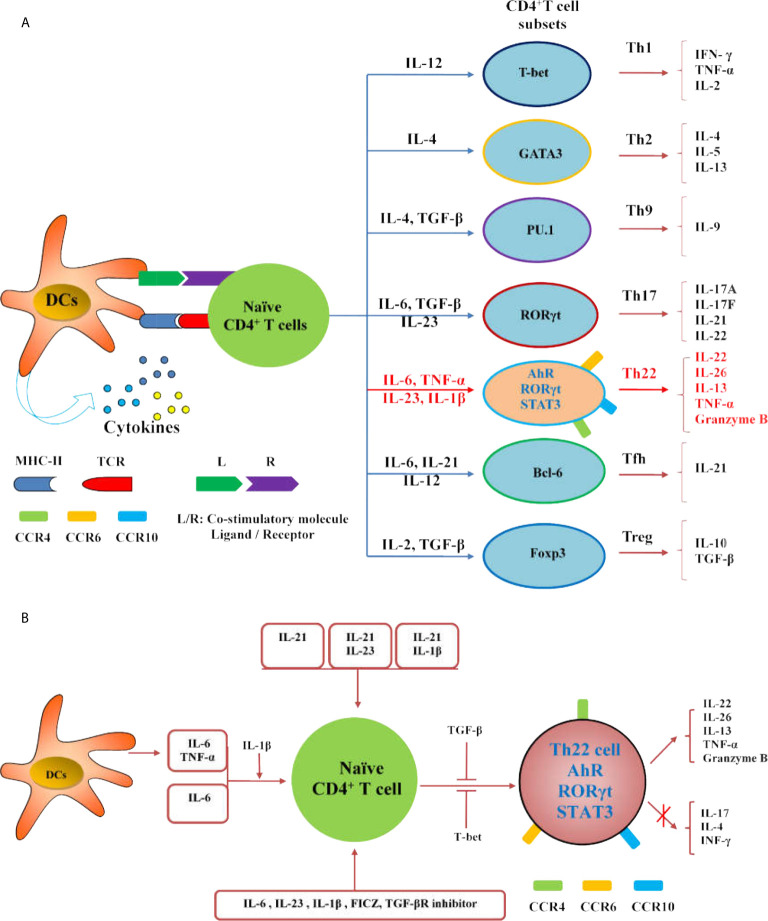
Regulation of Th22 cells differentiation. **(A)** The diagram illustrates the differentiation of Th cell subsets from naïve CD4^+^ T cells. **(B)** IL-21, IL-21 combined with IL-23 or IL-1β can induce the differentiation and IL-22 expression of Th22 cells; IL-6 and TNF-α secreted by DCs or external IL-6 or IL-6 and TNF- α can promote the differentiation of naïve CD4^+^T cells into Th22 cells, and IL-1β promotes the differentiation; IL-6, IL-23, IL-1β, FICZ and TGF-βR inhibitor can promote the differentiation of naïve CD4^+^T cells into Th22 cells. Both TGF-β and T-bet inhibit the expression of IL-22 in Th22 cells.

**Figure 3:**
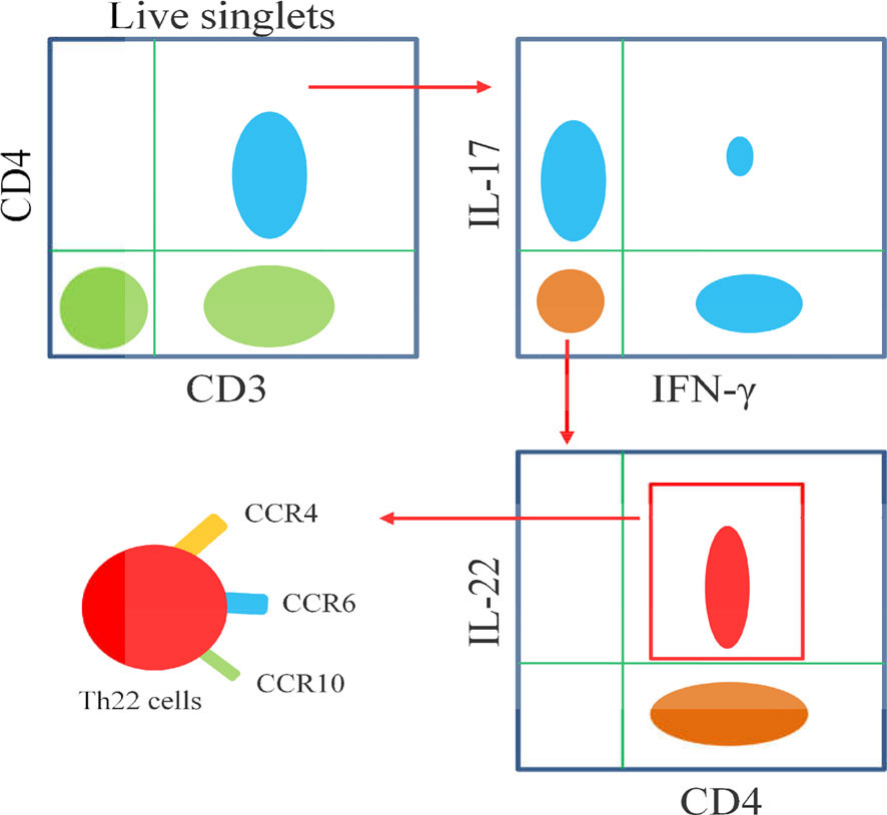
Schematic diagram illustrates the flow cytometric analysis of Th22 cells. Th22 cells are identified as CD3^+^CD4^+^IL-17^-^IFN-γ^-^IL-22^+^, as well as high expressions of CCR4,CCR6 and CCR10.

The differentiation of Th22 cells is regulated by many factors,which are different from other CD4^+^T cell subsets including Th1, Th17 and Tfh cells (**Figures 2A, B**). Both IL-6 and TNF-α can induce the differentiation of Th22 cells, IL-6 alone drives the differentiation of naïve CD4^+^T cells into Th22 cells. TNF-α further promotes the differentiation of Th22 cells induced by IL-6, while a high dose of TGF-β inhibits the differentiation of Th22 cells (10). In the presence of anti-IL-4/IFN-γ, IL-17A secretion still occurs when combined with IL-6,IL-23, IL-1β and 6-formylindolo (3, 2-b) carbazole (FICZ). The TGF-βR inhibitor (galunisertib) effectively inhibits the production of IL-17Abutdoes not affect the secretion of IL-22. Under this culture condition, the levels of IL-13 and granzyme B also increase significantly. Under this culture condition,the levels of granzyme B and IL-13 also increased significantly (16). IL-21 alone or in combination with IL-1β or IL-23 can induce Th22 cell differentiation and IL-22 expression (52). Many reports show that differentiated Th22 cells can be identified by integrating cell surface markers (CCR4^+^, CCR6^+^ and CCR10^+^) or its correlated intracellular cytokines including IL-22^+^, IL-17^-^ and IFN-γ^-^ **(** **Figure 3** **)** (15–19).

In addition,plasmacytoid-like dendritic cells (pDCs) could induce Th22 cells more strongly than conventional dendritic cells (cDCs), and both of them release high concentrations of TNF-α and IL-6 after activation. Blocking TNF-α and IL-6 inhibited 70% of Th22 cells in culture, which indicates that DCs may promote Th22 differentiation in both direct and indirect ways (10). Foreign antigens, such as microorganisms,can also activate DCs. After stimulation by the serotypes of *A. actinomycetemcomitans*, the levels of AhR and IL-22 in T lymphocytes were increased,and the levels of TNF-α and IL-6 in DCs were significantly increased. This type of actinomycete can trigger the polarization of Th22 cells, which may be an important part of the subgingival biofilm (53). Langerhans cells (LCs) from the human epidermis and dermis can also induce naïve CD4^+^T cells and peripheral blood T cells to differentiate into Th22 cells,and the effect of LCs on the epidermis is stronger than that of dermal DCs (54). Endogenous TLR4 ligands stimulate keratinocytes to secrete IL-23 which activates DCs and induces the differentiation of Th22 cells and IL-22 production (55). Activated B cells and initial T cells significantly inhibit the expression of RORγt and the production of IL-17, but significantly increase the differentiation of Th22 cells and the production of IL-22 by cultured Th17 cells *in vitro*. Further *in vivo* experiments showed that MRL/lpr lupus mice treated with activated B cells exhibited reduced levels of anti-dsDNA antibody and urinary protein. Meanwhile, Th17 cell differentiation was inhibited and Th22 cell differentiation was enhanced in these mice (56).

AhR is an important transcription factor in the differentiation of Th22 cells. However, the expression of IL-22 in naïve CD4^+^T cells was reduced in the absence of RORc (γt) but increased in Tbx21-deficient cells. Therefore, RORγt is considered a positive regulator of Th22, while T-bet is considered a negative regulator (16). Runt-related transcription factor 3 (RUNX3) is a runt-domain family transcription factor. The number of Th22 cells decreased significantly with the inhibition of RUNX3 (57). Recent studies have shown that miR-31 mimic transfection can increase the levels of AhR and IL-22 and promote the differentiation of Th22 cells in coronary heart disease. Overexpression of miR-31 promotes the differentiation of Th22 cells by inhibiting the BTB domain and CNC homolog-2 (Bach2) pathway (58). Medroxyprogesterone acetate (MPA) can also enhance the Th22 cell response and decrease the expression of Th1 and Th17 cell signature genes by activating AhR signaling, thus affecting susceptibility to inflammatory diseases and infectious diseases (59).

## Th22 Cells in Autoimmune Diseases

Recent studies have shown that Th22 cells play a regulatory role in the initiation and development of many diseases, such as kidney disease (60), cardiovascular disease (61), tumors (62) and infectious diseases (63). IL-22, the main effector of Th22 cells,also exerts different functions in different autoimmune diseases. Cytokines of the IL-10 family mainly act on interstitial cells and tissue epithelial cells, which can promote the proliferation and repair of tissues and organs, protect the integrity of the barrier, and play a patrolling role (23). These cytokines have both proinflammatory and anti-inflammatory functions. Here, the roles of Th22/IL-22 in the pathogenesis of autoimmune diseases are reviewed (**Table 1**).

**Table 1:** Dual role of Th22/IL-22 in autoimmune diseases.

Disease	Mechanism of Th22 cells	Th22 frequency	IL-22 serum level	References
***Pathogenic***				
*RA*	*Promote osteoclast differentiation, induce osteoclast formation by p38MAPK/NF-κB and JAK2/STAT3 signaling*	↑	↑	(20, 64, 65)
*SLE*	*Positively correlated with Th17 cells, correlated with the disease activity index and the severity index*	*↑*	↑	(66–68)
		*↓*	*↓*	(69–71)
*Ps*	*Activate keratinocyte overproliferation, induce dermatitis and acanthosis by activating the STAT3-mediated IL-23 pathway*	↑	↑	(72–74)
*MS*	*Activate the NF-κB pathway, inhibit Foxp3 expression,promote oligodendrocyte apoptosis*	↑	↑	(75, 76)
*ITP*	*Positively correlated with Th1/Th17/Tfh cells*	↑	↑	(77–79)
*IgAN*	*Activate STAT3 and JAK signaling pathways, regulate renal fibrosis through the ERK,AKT and p38 signaling pathways*	↑	↑	(80, 81)
*AIH*	*Th1/Th17/Th22 imbalance with Treg*	↑	↑	(82)*[A]*
*AITD*	*Secrete proinflammatory cytokines, such as IL-22 and IL-6*	↑	↑	(83–86)
*SSc*	*Express massive fibroblast growth factor, promote the response of skin fibroblasts to TNF-α*	↑	↑	(87–89)
*AS*	*Positively correlate with Th17 cells*	↑	↑	(20)
		*—*	↑	(90)
*Vasculitis*	*Involved in GCA B cell proliferation, differentiation and arterial remodeling*	*	↑	(91)
***Protective***				
*MS*	*Severity of EAE was reduced in IL-22BP deficient mice*	*	↑	(92)*[A]*
*MG*	*IL-22 level was negatively correlated with serum anti-ACHR antibody level*	*	*↓*	(93)
		*—*	*	(94)

”↑” represents an increase compared to the healthy control; “↓” represents a decrease compared to the healthy control; “—” indicates similar to the healthy control; “*” indicates not mentioned; “[A]” indicates animal model; non an notated references include human studies.

RA, rheumatoid arthritis; SLE, systemic lupus erythematosus; Ps, psoriasis; MS, multiple sclerosis; ITP, immune thrombocytopenia; IgAN, immunoglobulin A nephropathy; AIH, autoimmune hepatitis; AITD, autoimmune thyroid diseases; SSc, systemic sclerosis; AS, ankylosing spondylitis; MG, myasthenia gravis.

### RA

RA is a common autoimmune disease that is characterized by uncontrolled joint inflammation, bone erosion and cartilage damage. The levels of Th22 cells, Th17 cells and IL-22 in patients with RA were significantly higher than those in healthy controls. The numbers of Th22 cells were positively correlated with the IL-22 levels. Moreover, the percentages of Th22 cells and Th17 cells were positively correlated with the disease activity score in 28 joints (DAS28) scores and C-reactive protein (CRP) levels in RA patients (20, 64, 65). Not only did the levels of IL-17 and IL-22 in plasma increase, but the levels of IL-17 and IL-22 in the subchondral bone marrow of patients with RA were also significantly higher than those in plasma. The percentage of Th1,Th17 and Th22 cells and the levels of IL-17 and IL-22 in bone marrow were also positively correlated with DAS28 (95). The frequency of Th22 and Th17 cells in peripheral blood and the levels of IL-22, IL-17 and IFN- γ in plasma were decreased in RA patients who received effective treatment with methotrexate and leflunomide, although significant changes were not observed in patients with nonresponsive RA (96).

However,there are different views on whether Th22/IL-22 is involved in T cell-mediated synovitis. Researchers have found that the disease severities of wild-type mice and IL-22-deficient mice are comparable to those of T cell-mediated arthritis,Th17 cells have a strong effect on synovial fibroblasts while targeting Th17 cells and IL-17A but not Th22/IL-22 has been suggested as a focus treatment for T cell-mediated synovial inflammation (97). Recently, IL-22 neutralization was shown to inhibit osteoclast formation. Th22 cells promote osteoclast differentiation by producing IL-22 and play an important role in bone destruction in patients with RA (98). High levels of IL-22 in synovial tissue induce the proliferation of synovial fibroblasts and produce chemokines to enhance the inflammatory response of synovial tissue (29). IL-22 also induces osteoclast formation by inducing the p38MAPK/NF-κB and JAK2/STAT3 signaling pathways in synovial fibroblasts. Moreover, Th22 cells migrate to synovial tissues, which might be associated with the high expression of C-C chemokine ligand 28 (CCL28) in RA patients (98). IL-22 induces osteoclast formation by p38MAPK/NF-κB and JAK2/STAT3 signaling in RA development (51). In an animal model of CIA, blocking chemokine receptors was able to effectively inhibit the progression of RA. After treatment with the selective CXCR3 antagonist AMG487, the percentage of Treg cells in CIA mice was increased while the percentages of Th1, Th17 and Th22 cells were decreased. The expression of T-bet, IL-17A, IL-22 and RORγt was down-regulated, and the expression of Foxp3 was up-regulated. Chemokine receptor antagonists have been suggested as an effective strategy for the treatment of RA (99). Current studies support the hypothesis that Th22/IL-22 plays a pathogenic role in RA pathogenesis, although this mechanism requires further study. Blocking IL-22 may serve as a novel effective therapeutic method for the treatment of the disease.

### SLE

SLE is another common autoimmune disease that is characterized by increased autoantibodies and immune disorders leading to tissue and organ damage. The role of Th22 cells in SLE is still controversial. Zhao et al. found that the plasma levels of IL-17A and IL-22 in patients with SLE were higher than those in healthy controls, and they showed that Th17 and IL-22 levels were positively correlated with the SLE disease activity index (SLEDAI), indicating that IL-22 and IL-22^+^CD4^+^ T cells play an important role in the pathogenesis of SLE (66). In 2014, the team further found that the levels of IL-22 and IL-22^+^CD4^+^ T cell before and after immunosuppressant and glucocorticoid (GC) treatment did not differ compared with the healthy controls (100). In 2017, this group also found that the levels of CCR6^+^ T cells, CCR6^+^Th22 cells and plasma IL-22 increased in SLE patients. The percentage of Th22 cells was positively correlated with the area of lupus erythematosus and the severity index (RCLASI) of the skin. The percentage of Th22 cells in SLE patients with renal damage was positively correlated with ESR, suggesting that CCR6^+^ Th22 cells may be a therapeutic target for SLE treatment (67). Defects in TGF-β1 signaling in patients with active SLE are also associated with the over-production of IL-22 (101). Yang et al. found that the IL-22 levels increased in MRL/LPR mice while treatment with anti-IL-22 monoclonal antibody significantly decreased the urinary protein, urea nitrogen and serum creatinine in these mice (68). Moreover, Th22 cells might be a better predictor of SLE tissue involvement than Th17 cells (102). Other studies have shown that the level of IL-22 is decreased in patients with SLE (69–71) and significantly lower in patients with primary SLE (70). Urinary IL-22 mRNA levels are decreased in SLE patients with proliferative glomerulonephritis. IL-22 mRNA can also be used to evaluate the activity of lupus nephritis (71). In Chinese SLE patients, IL-22 gene polymorphisms may increase susceptibility to SLE by reducing the expression of IL-22 (103). In addition, recent studies have shown that activated B cells suppress the development of lupus by promoting Th22 cell differentiation and inhibiting Th17 (56). These results show that both Th22 cells and IL-22 levels are related to SLE; however, the exact mechanism is still unclear. Moreover,these studies show that IL-22 levels differ at different stages of SLE (70) and indicate its involvement in tissue inflammation and damage to different organs (102). The currently available results suggest the complexity of IL-22 and the heterogeneity of SLE, which needs to be further explored.

### PSORIASIS

Psoriasis is a chronic inflammatory autoimmune disease mediated by T cells, and it is characterized by the abnormal proliferation of keratinocytes. IL-22 is considered an activator of keratinocyte over-proliferation (104, 105). The levels of Th22 cells and plasma IL-22 in patients with psoriasis are increased and positively correlated with the severity of the disease (72–74). High levels of IL-22 can induce the expression of antimicrobial proteins (AMPs), antimicrobial peptides such as S100A7,S100A8, S100A9 and β-defensin, and neutrophil chemoattractants CXCL8, CXCL5 and CXCL1 in the epidermis (106, 107). It can also inhibit keratinocyte differentiation, interfere with the normal skin healing process, and induce the production of MMPs, which is conducive to extracellular tissue degradation (108, 109). IL-22 also induces dermatitis and acanthosis by activating the STAT3-mediated IL-23 pathway (9, 110). In addition, IL-22 neutralizing antibody treatment decreased antimicrobial peptide levels and inhibited disease development in a psoriasis mice model, suggesting the therapeutic potential of IL-22 inhibitors in psoriasis (111).

Psoriasis is characterized by recurrent lesions in the same anatomic area. New lesions appear in areas that have healed after successful treatment because even if the psoriatic plaque disappears, tissue resident memory cells (TRMs) are still present in the skin (105, 112). In the active stage of psoriasis,the expression of IFN-γ, IL-17A and IL-22 in CD4^+^ and CD8^+^ T cells in the epidermis is increased. IL-22 is mainly produced by CD4^+^ epidermal T cells, where it activates keratinocytes and leads to acanthosis. IL-17A is mainly produced by epidermal CD8^+^ T cells, and it drives keratinocytes to participate in the recruitment of neutrophils and produces chemokines and proinflammatory cytokines (113). TNF-α promotes the differentiation of Th22 cells, while TNF-α blockade has achieved satisfactory results in the treatment of psoriasis patients. However, Th22 cells in the healed skin epidermis still produce IL-22 after 6 years of remission. Therefore, Th22 cells play an important role in the memory of psoriatic relapse (113). Previous findings suggest that IL-22 in the psoriatic epidermis is produced by IL22^+^IL-17^+^ Th17 cells. Recent studies show that there is no significant correlation between the expression of IL-17A and IL-22 in psoriasis, which does not support the existence of double-secreted IL-17A/IL-22 Th17 cells (114).

### MS

MS is an autoimmune disease of the central nervous system (CNS), and it is characterized by the loss of the axonal myelin sheath and inflammation of the CNS, showing clinical symptoms such as muscle spasm and paralysis. Proinflammatory cytokines such as IL-22, IL-17, TNF-α and IFN-γ are involved in MS pathogenesis through multiple signaling pathways. The proportion of Th22 cells (75, 76) and the level of IL-22 in the serum of patients with MS increased (75, 76, 115–121), and the level of IL-22 in recurrent stage was significantly higher than that in progressive stage and remission stage (115, 116, 119, 122). The level of IL-22 in cerebrospinal fluid is also increased, and IL-22 increases the survival rate of brain astrocytes. The IL-22 receptor subunit IL-22R is mainly expressed on astrocytes (119), suggesting that astrocytes may play an important role in IL-22-mediated pathological changes in MS. IL-22 also inhibits the expression of Foxp3 by activating the NF-κB pathway and promotes the expression of Fas in oligodendrocytes, which leads to apoptosis of oligodendrocytes (123). Large-scale studies of 5019 MS patients in Norway and Sweden by Beyenet et al. identified IL-22RA2 as a risk gene for MS (124). The high expression of T-bet and CCR6 in Th22 cells of MS patients suggests that Th22 cells may migrate to the central nervous system. The infiltration of Th22 cells leads to an increase in T cell infiltration and contributes to the destruction of the blood-brain barrier (76, 125). In addition, the resistance to IFN-β therapy in MS patients may be related to the low expression of IFN receptor 1 on the surface of Th22 cells, and similar findings have been observed in the MS animal model of EAE (115).

A study in 2014 suggested that IL-22 played a protective role in CNS inflammation (126). The severity of inflammation was positively correlated with the level of IL-22BP in the cerebrospinal fluid of patients with MS. IL-22BP has a pathogenic effect on EAE in both mice and rats, and IL-22BP is theoretically an antagonist of IL-22. IL-22BP has been proposed to reduce IFN-γ produced by brain-derived T cells in lymph nodes (92). Previous studies showed that the histopathological features of EAE in IL-22-deficient mice were comparable to those in wild-type mice (14). Hannes et al. conducted EAE experiments on wild-type mice, IL-22 deficient mice, IL-22BP deficient mice, and IL-22 and IL-22BP double deficient mice and concluded that the loss of control of IL-22 signal in IL-22BP deficient mice reduced the severity of EAE,which supported the protective effect of IL-22 in MS; thus, they suggested that IL-22BP could be used as a new target for MS (92).

### ITP

ITP is an autoimmune disease that is characterized by increased destruction and decreased production of platelets (127). The frequency of Th22 cells and the level of plasma IL-22 in patients with ITP were significantly higher than those in the control group (77, 78, 128). The increase in plasma IL-22 levels in ITP patients was reported to be associated with the dysregulation of Th1 and Th22 cells (128). Furthermore, Th22 cells in ITP patients were positively correlated with Th1 and Th17 cells (77, 79), and patients treated with high-dose dexamethasone (HD-DXM) exhibited significantly decreased frequencies of Th1 and Th22 cells and plasma concentrations of IL-22 (78). The level of IL-22 was also increased in pediatric ITP patients compared with healthy populations (129). A recent study showed that the frequencies of Th22, Th17, Tfh and Th1 cells in the bone marrow of ITP patients were significantly higher than those in the control group; moreover, the frequency of Th22 cells in bone marrow was significantly higher than that in peripheral blood (130). Notably, ITP patients with negative autoantibodies showed a higher percentage of Th22 cells than patients with positive detection of autoantibodies (77). In summary, Th22 plays a proinflammatory effect in ITP and acts synergistically with Th1/Th17 and Tfh cells. Th22 cells play an important role in the pathophysiological process of ITP patients. Therefore, blocking IL-22 may serve as a potential therapeutic target for treating ITP patients.

### IGAN

IgAN is the most common primary glomerular disease characterized by inflammatory cell infiltration and IgA deposition in the mesangial area of the glomerulus, which is an important cause of renal failure (131). Although the exact pathogenesis is still unclear, the involvement of T cells has been confirmed. IgAN has a higher proportion of circulating Th2, Th17, Th22, Tfh and γδ T cells, but a lower proportion of Treg and Th1 cells (80). Peng et al. also showed that Th17 and Th22 cell frequencies in peripheral blood and plasma IL-22 levels were significantly increased in patients with IgAN, and Th22 cells were positively correlated with Th17 cells and plasma IL-22 levels. In addition, compared with IgAN patients without proteinuria, IgAN patients with proteinuria showed a higher percentage of Th22 cells (81). Th22 cells in patients with IgAN are higher than those in healthy controls, and the percentage of increase is positively correlated with the degree of kidney disease in patients with IgAN. Moreover,tonsillitis aggravates the over-expression of Th22 cells and the chemokines CCL20, CCL22 and CCL27 and aggravates kidney damage in IgAN (132). Xiao et al. confirmed that streptococcal infection can increase the chemotaxis of Th22 cells and aggravate kidney inflammation (133). Chronic inflammation is a common sign of chronic fibrosis,and fibrosis is one of the common pathological changes of IgAN. IL-22 binds to kidney IL-22R1 to activate STAT3, JAK and other signaling pathways, and regulate renal fibrosis through the ERK, Akt, and p38MAPK signaling pathways. Therefore,Th22 cells promote renal fibrosis in IgAN (134, 135). Treatment is mainly reflected in the reduced chemotaxis of Th22 cells. After treatment with cordyceps (CS), dexamethasone and losartan, the frequency of Th22 cells in the IgAN mouse model decreased and the expression of CCR10, CCL27 and IL-22 was also significantly reduced (60, 136). The above treatments all regulate the chemotaxis of Th22 cells to inhibit inflammation and improve renal function in patients. Acteoside (the main component of Rehmannia glutinosa) can also inhibit Th22 cell proliferation and inhibit Th22 cell chemotactic factors to regulate Th22 cell chemotaxis (137). In addition, the IL-22R1 gene polymorphism is genetically associated with the development of childhood IgA nephropathy (138).

### AIH

AIH is a chronic autoimmune inflammatory liver disease that is characterized by high autoantibodies, high liver enzyme levels,and liver damage (139). Hepatocytes are one of the target cells of IL-22. IL-22 has dual effects on hepatocytes: protecting hepatocytes and inducing acute phase proteins. IL-22 acts on liver progenitor cells (LPCs), which is beneficial for liver reconstruction after injury. In human and mouse chronic HBV-infected livers, IL-22 promotes hepatocyte proliferation through the STAT3 pathway (140, 141). Patients with drug-induced liver injury (DILI) have increased intrahepatic and peripheral Th22 cells and IL-22 levels, and the liver IL-22 level is positively correlated with regeneration. Th22/IL-22 has a hepatoprotective effect in DILI (142). Studies have indicated that the increase in Th22/IL-22 is related to the severity of hepatitis B virus-related chronic liver failure (HBV-ACLF) and suggested that Th22/IL-22 can be used as a biomarker for the prognosis of HBV-ACLF (143). Few studies have focused on the pathogenesis of Th22/IL-22 in AIH. Studies have shown that compared with healthy controls, the serum levels of IL-6,IL-10,IL-17F, IL-21, IL-23 and TNF-α in AIH are significantly increased while those of IL-22 and IL-17A are not. According to the grouping of immunoserological markers, the cytokines of type 2 AIH patients are characterized by elevated levels of IL-21 and IL-22 (144). The mouse experimental autoimmune hepatitis (EAH) model and AIH patients present reduced serum IL-10 and Treg levels. The number of Th22, Th17 and Th1 cells and the number of corresponding cytokines IL-22, IL-17A and IFN-γ were all reduced. Moreover, the number of Tregs was negatively correlated with the number of Th22, Th17 and Th1 cells and cytokine levels. More interestingly, the serum IL-22 and IL-17A levels were positively correlated with liver injury in patients with AIH, suggesting that the imbalance between Th1/Th17/Th22 and Treg cells may be involved in the process of AIH (82).

### AITD

AITD mainly includes Graves’ disease (GD) and Hashimoto’s thyroiditis (HT). GD manifests as hyperthyroidism caused by the overproduction of thyroid hormone, while HT manifests as hypothyroidism (145). T cell dysfunction and/or corresponding cytokine abnormalities cause the destruction of immune tolerance, which leads to abnormal immune responses in AITD. Research on Th22/IL-22 in AITD is also limited. The study by Peng et al. showed that the percentage of Th17 and Th22 cells and plasma IL-17 and IL-22 in GD patients were increased and positively correlated with serum TSAb levels (83, 84). GD patients not only have higher Th22 cell frequencies and serum IL-22 levels than healthy people but also have higher IL-22 mRNA and AhR expression, whereas HT patients do not show an increase in Th22/IL-22 (146). However, different results have also been presented. Bai et al. found that the circulating Th22 cell level of HT patients was significantly higher than that of the healthy control group and the GD patient group, and was positively correlated with the serum IL-22 level and thyroid peroxidase antibody (TPOAb) titer. Under TNF-α and IL-6 stimulation,the T lymphocytes of HT patients showed an enhanced ability to differentiate into Th22 cells *in vitro* (85). Ruggeri et al. also believed that serum IL-22 levels in untreated,newly diagnosed HT patients were higher than those in healthy controls (86). The level of Th22/IL-22 in AITD patients is elevated,and Th22 cells may participate in the pathogenesis of AITD by secreting IL-22, IL-6 and other proinflammatory cytokines; however, the exact mechanism remains to be further confirmed.

### MG

MG is an autoimmune disease that produces anti-acetylcholine receptor (ACHR) autoantibodies and neuromuscular transmission disorders and manifests as skeletal muscle fatigue and weakness (147). Thymectomy (TE) represents one of the treatment methods. The frequency of Th22 cells in patients treated with TE was not significantly different from that of the healthy controls. After TE surgery, the frequency of Th22 cells was significantly reduced (93). Studies have also shown that the levels of IL-17 mRNA in PBMC and IL-17 concentrations in serum increase while levels of IL-22 mRNA and serum IL-22 decrease in MG patients. In addition, the level of serum IL-22 is negatively correlated with the level of serum anti-ACHR antibody, suggesting that IL-22 plays a protective role in MG (94). A recent study showed that the levels of IL-22 in the PBMCs of MG patients did not differ from those in the control group (148). Thus, further research is required to clarify the specific role of Th22/IL-22 in MG.

## Other Autoimmune Diseases

SSc is an autoimmune connective tissue disease with skin and visceral organ fibrosis due to excessive deposition of extracellular matrix and vascular lesions (149). The increased frequency of circulating Th22 cells is positively associated with pulmonary interstitial disease in SSc patients (87). Moreover, the excessive production of IL-22 in injured skin was independent of IL-17 (88). Other studies have shown that IL-22 can enhance the response of fibroblasts to TNF-α, promote the inflammatory phenotype of fibroblasts, and enhance the ability of TNF-activated keratinocytes to stimulate fibroblasts (89). Th22 cells express massive amounts of fibroblast growth factor, suggesting that targeting the IL-22 signaling pathway may be effective for preventing fibrogenesis (150). Th22/IL-22 may be related to skin and visceral fibrosis in patients with SSc. Primary Sjogren’s syndrome (pSS) is a chronic autoimmune disease that is characterized by lymphocyte infiltration in lacrimal and salivary glands, and it also presents elevated IL-22 levels (43, 151, 152). IL-22 plays a proinflammatory role in pSS pathogenesis and promotes salivary gland inflammation at an early stage (152, 153). IL-22 is predominantly secreted by Th17 and NKp44^+^ NK cells in pSS patients (152). Studies have shown that the frequency of Th22 cells in the peripheral blood of ankylosing spondylitis (AS) patients is increased (20, 154), while other studies have shown that there is no difference in IL-22^+^CD4^+^ and IL-22^+^CD8^+^ T cells between AS patients and healthy controls; however, the secretion of IL-22 by circulating mucosal-associated invariant T (MAIT) cells is increased in AS patients (90). A number of types of vasculitis have been identified, and several studies have focused on Th22/IL-22 in vasculitis. A study by Zerbini et al. showed that the levels of IL-22 and IL-22R1 were higher in giant cell arteritis (GCA) patients who were confirmed to be positive for temporal artery biopsy (TAB) than in TAB-negative patients and normal controls. IL-22 is expressed in spindle cells and infiltrating immune cells, while IL-22R1 is expressed in endothelial cells; moreover, IL-22 is involved in B cell proliferation, differentiation and arterial remodeling of GCA (91).

## Therapeutic Targeting of IL-22

Previous investigations have shown a critical role of IL-22 during the pathogenesis of autoimmune diseases. Preclinical studies indicate that IL-22 may serve as a promising therapeutic target for treating autoimmune diseases. Th22 cells promote osteoclast differentiation while neutralization of IL-22 inhibits osteoclast formation, suggesting that blocking IL-22 could be effective in suppressing bone destruction in RA patients (98). Inhibition of IL-22 by neutralization antibodies has been shown to reduce the expression of chemotactic factors, decrease antimicrobial and hyperproliferative responses of keratinocytes, and prevent the development of imiquimod-induced psoriasis from skin inflammation (111). It has been reported that treatment with cordyceps sinensis, dexamethasone and losartan improves kidney functions associated with a reduction of Th22 cells in mice with IgA nephropathy, suggesting that these drugs may exert their protective effects through modulating Th22 cells (60, 136).

Currently,the safety, tolerability and therapeutic effects of Fezakinumab (ILV-094), a human monoclonal antibody that directly binds to IL-22, have been examined in atopic dermatitis patients in severalclinical studies (155). A small scale randomized,double-blind, phase 2a clinical trial involving 60 patients with moderate-to-severe atopic dermatitis has shown that Fezakinumab is well-tolerated with sustained clinical improvements after last drug dosing. Fezakinumab treatment has shown significant clinical improvements *versus* placebo in patients with severe disease as reflected by significant reductions of SCORing of Atopic Dermatitis (SCORAD) scores and Investigator Global Assessment (156). Furthermore, transcriptomic and immunohistochemistry analyses reveal that Fezakinumab has profound effects on multiple inflammatory pathways in these patients (157). Fezakinumab broadly decreases immune activation in skin tissues and reduces overall inflammatory burden and epidermal pathologic characteristics. The treatment effects of Fezakinumab are particularly evident in patients with high IL-22 baseline expression, suggesting that a precision medicine-based approach might be needed for improving therapeutic outcomes in patients with atopic dermatitis.

It has been revealed that IL-22 exerts protective roles in certain diseases. A phase II study (NCT02406651) is undergoing to investigate the therapeutic effects of recombinant IL-22 for GVHD after bone marrow transplantation (158). Other potential strategies including the modulation of chemotaxis of Th22 cells,administration of AhR agonists to enhance IL-22 expression,and application of IL-22BP are under investigation. Available results suggest that Th22 cells are involved in autoimmune pathogenesis through multiple effector functions, including the production of various cytokines, such as IL-22, IL-13 and IL-26. Moreover, Th22 cells may have close interactions with Th1, Th2 and Th17 cells during disease progression. Further investigations on the safety,tolerability and therapeutic effects of agents targeting Th22/IL22 pathway are needed for the effective treatment of autoimmune diseases.

## Conclusion

Th22 cells and IL-22play diverse roles in the development of autoimmune diseases and have both proinflammatory and anti-inflammatory functions. Th22/IL-22 plays a pathogenic role in most autoimmune diseases, while IL-22 has been shown to have a protective effect in many other diseases involving skin and mucosal barrier. Thus, the function of IL-22 varies depending on the cellular source, types of inflammatory response, the affected tissue (mucosa or solid organ), and the concentration and duration of IL-22 itself in local environment. Emerging evidence supports the notion that Th22 cells may serve as therapeutic targets for autoimmune diseases. However, further studies are needed to elucidate the mechanisms of Th22 cells in disease pathogenesis and validate the therapeutic potential of targeting Th22 cells for the treatment of autoimmune diseases.

## Author Contributions

QJ and DC drafted the manuscript and designed the figures. GY and FX reviewed the manuscript structure and ideas. DC, JX, SW, and LL conceived the topic and revised the manuscript. All authors contributed to the article and approved the submitted version.

## Funding

This work was supported by the National Natural Science Foundation of China (Grant Nos. 81871709, 81971994, 82071817, 91846103, 31711530025 and 81771759), Funding for Chongqing International Institute for Immunology (2020YJC10), Hong Kong Research Grants Council General Research Fund (17103821, 17113319) and Theme-Based Research Scheme (T12-703/19R), Zhejiang Provincial Key Research and Development Program (Grant No. 2020C03032).

## Conflict of Interest

The authors declare that the research was conducted in the absence of any commercial or financial relationships that could be construed as a potential conflict of interest.

## References

[1] Wang W Sung N Gilman-Sachs A Kwak-Kim J. T Helper (Th) Cell Profiles in Pregnancy and Recurrent Pregnancy Losses: Th1/Th2/Th9/Th17/Th22/Tfh Cells. Front Immunol (2020) 11:2025. 10.3389/fimmu.2020.02025 32973809 PMC7461801

[2] Ruterbusch M Pruner KB Shehata L Pepper M. In Vivo CD4(+) T Cell Differentiation and Function: Revisiting the Th1/Th2 Paradigm. Annu Rev Immunol (2020) 38:705–25. 10.1146/annurev-immunol-103019-085803 32340571

[3] Chatzileontiadou DSM Sloane H Nguyen AT Gras S Grant EJ. The Many Faces of CD4(+) T Cells: Immunological and Structural Characteristics. Int J Mol Sci (2020) 22(1):73. 10.3390/ijms22010073 PMC7796221 33374787

[4] Xiao F Han M Rui K Ai X Tian J Zhang W. New Insights Into Follicular Helper T Cell Response and Regulation in Autoimmune Pathogenesis. Cell Mol Immunol (2021) 18(6):1610–2. 10.1038/s41423-021-00688-7 PMC8166848 33972739

[5] Mosmann TR Cherwinski H Bond MW Giedlin MA Coffman RL. Two Types of Murine Helper T Cell Clone. I. Definition According to Profiles of Lymphokine Activities and Secreted Proteins. J Immunol (1986) 136(7):2348–57. 2419430

[6] Park H Li Z Yang XO Chang SH Nurieva R Wang YH. A Distinct Lineage of CD4 T Cells Regulates Tissue Inflammation by Producing Interleukin 17. Nat Immunol (2005) 6(11):1133–41. 10.1038/ni1261 PMC1618871 16200068

[7] Harrington LE Hatton RD Mangan PR Turner H Murphy TL Murphy KM. Interleukin 17-Producing CD4+ Effector T Cells Develop *via* a lineage distinct from the T helper type 1 and 2 lineages. Nat Immunol (2005) 6(11):1123–32. 10.1038/ni1254 16200070

[8] Gurney AL. IL-22, a Th1 Cytokine That Targets the Pancreas and Select Other Peripheral Tissues. Int Immunopharmacol (2004) 4(5):669–77. 10.1016/j.intimp.2004.01.016 15120651

[9] Zheng Y Danilenko DM Valdez P Kasman I Eastham-Anderson J Wu J. Interleukin-22, a T(H)17 Cytokine,Mediates IL-23-Induced Dermal Inflammation and Acanthosis. Nature (2007) 445(7128):648–51. 10.1038/nature05505 17187052

[10] Duhen T Geiger R Jarrossay D Lanzavecchia A Sallusto F. Production of Interleukin 22 But Not Interleukin 17 by a Subset of Human Skin-Homing Memory T Cells. Nat Immunol (2009) 10(8):857–63. 10.1038/ni.1767 19578369

[11] Dumoutier L Louahed J Renauld JC. Cloning and Characterization of IL-10-Related T Cell-Derived Inducible Factor (IL-TIF), a Novel Cytokine Structurally Related to IL-10 and Inducible by IL-9. J Immunol (2000) 164(4):1814–9. 10.4049/jimmunol.164.4.1814 10657629

[12] Xie MH Aggarwal S Ho WH Foster J Zhang Z Stinson J. Interleukin (IL)-22,a Novel Human Cytokine That Signals Through the Interferon Receptor-Related Proteins CRF2-4 and IL-22r. J Biol Chem (2000) 275(40):31335–9. 10.1074/jbc.M005304200 10875937

[13] Wolk K Sabat R. Interleukin-22: A Novel T- and NK-Cell Derived Cytokine That Regulates the Biology of Tissue Cells. Cytokine Growth Factor Rev (2006) 17(5):367–80. 10.1016/j.cytogfr.2006.09.001 17030002

[14] Kreymborg K Etzensperger R Dumoutier L Haak S Rebollo A Buch T. IL-22 is Expressed by Th17 Cells in an IL-23-Dependent Fashion, But Not Required for the Development of Autoimmune Encephalomyelitis. J Immunol (2007) 179(12):8098–104. 10.4049/jimmunol.179.12.8098 18056351

[15] Eyerich S Eyerich K Pennino D Carbone T Nasorri F Pallotta S. Th22 Cells Represent a Distinct Human T Cell Subset Involved in Epidermal Immunity and Remodeling. J Clin Invest (2009) 119(12):3573–85. 10.1172/JCI40202 PMC2786807 19920355

[16] Plank MW Kaiko GE Maltby S Weaver J Tay HL Shen W. Th22 Cells Form a Distinct Th Lineage From Th17 Cells In Vitro With Unique Transcriptional Properties and Tbet-Dependent Th1 Plasticity. J Immunol (2017) 198(5):2182–90. 10.4049/jimmunol.1601480 PMC5367520 28100680

[17] Yang P Qian FY Zhang MF Xu AL Wang X Jiang BP. Th17 Cell Pathogenicity and Plasticity in Rheumatoid Arthritis. J Leukocyte Biol (2019) 106(6):1233–40. 10.1002/jlb.4ru0619-197r 31497905

[18] Mousset CM Hobo W Woestenenk R Preijers F Dolstra H van der Waart AB. Comprehensive Phenotyping of T Cells Using Flow Cytometry. Cytometry A (2019) 95(6):647–54. 10.1002/cyto.a.23724 30714682

[19] Trifari S Kaplan CD Tran EH Crellin NK Spits H. Identification of a Human Helper T Cell Population That has Abundant Production of Interleukin 22 and is Distinct From T(H)-17,T(H)1 and T(H)2 Cells. Nat Immunol (2009) 10(8):864–71. 10.1038/ni.1770 19578368

[20] Zhang L Li YG Li YH Qi L Liu XG Yuan CZ. Increased Frequencies of Th22 Cells as Well as Th17 Cells in the Peripheral Blood of Patients With Ankylosing Spondylitis and Rheumatoid Arthritis. PloS One (2012) 7(4):e31000. 10.1371/journal.pone.0031000 22485125 PMC3317658

[21] Gittler JK Shemer A Suarez-Farinas M Fuentes-Duculan J Gulewicz KJ Wang CQ. Progressive Activation of T(H)2/T(H)22 Cytokines and Selective Epidermal Proteins Characterizes Acute and Chronic Atopic Dermatitis. J Allergy Clin Immunol (2012) 130(6):1344–54. 10.1016/j.jaci.2012.07.012 PMC3991245 22951056

[22] Nograles KE Zaba LC Shemer A Fuentes-Duculan J Cardinale I Kikuchi T. IL-22-Producing “T22” T Cells Account for Upregulated IL-22 in Atopic Dermatitis Despite Reduced IL-17-Producing TH17 T Cells. J Allergy Clin Immunol (2009) 123(6):1244–52.e2. 10.1016/j.jaci.2009.03.041 19439349 PMC2874584

[23] Ouyang W O’Garra A. IL-10 Family Cytokines IL-10 and IL-22: From Basic Science to Clinical Translation. Immunity (2019) 50(4):871–91. 10.1016/j.immuni.2019.03.020 30995504

[24] Wolk K Kunz S Witte E Friedrich M Asadullah K Sabat R. IL-22 Increases the Innate Immunity of Tissues. Immunity (2004) 21(2):241–54. 10.1016/j.immuni.2004.07.007 15308104

[25] Xu W Presnell SR Parrish-Novak J Kindsvogel W Jaspers S Chen Z. A Soluble Class II Cytokine Receptor,IL-22RA2,is a Naturally Occurring IL-22 Antagonist. Proc Natl Acad Sci USA (2001) 98(17):9511–6. 10.1073/pnas.171303198 PMC55483 11481447

[26] Wolk K Kunz S Asadullah K Sabat R. Cutting Edge: Immune Cells as Sources and Targets of the IL-10 Family Members? J Immunol (2002) 168(11):5397–402. 10.4049/jimmunol.168.11.5397 12023331

[27] Hansson M Silverpil E Linden A Glader P. Interleukin-22 Produced by Alveolar Macrophages During Activation of the Innate Immune Response. Inflammation Res (2013) 62(6):561–9. 10.1007/s00011-013-0608-1 23474919

[28] Zindl CL Lai JF Lee YK Maynard CL Harbour SN Ouyang W. IL-22-Producing Neutrophils Contribute to Antimicrobial Defense and Restitution of Colonic Epithelial Integrity During Colitis. Proc Natl Acad Sci USA (2013) 110(31):12768–73. 10.1073/pnas.1300318110 PMC3732935 23781104

[29] Ikeuchi H Kuroiwa T Hiramatsu N Kaneko Y Hiromura K Ueki K. Expression of Interleukin-22 in Rheumatoid Arthritis: Potential Role as a Proinflammatory Cytokine. Arthritis Rheum (2005) 52(4):1037–46. 10.1002/art.20965 15818686

[30] Simonian PL Wehrmann F Roark CL Born WK O’Brien RL Fontenot AP. γδ T Cells Protect Against Lung Fibrosis *via* IL-22. J Exp Med (2010) 207(10):2239–53. 10.1084/jem.20100061 PMC2947077 20855496

[31] Dudakov JA Hanash AM van den Brink MR. Interleukin-22: Immunobiology and Pathology. Annu Rev Immunol (2015) 33:747–85. 10.1146/annurev-immunol-032414-112123 PMC4407497 25706098

[32] Cochez PM Michiels C Hendrickx E Van Belle AB Lemaire MM Dauguet N. AhR Modulates the IL-22-Producing Cell Proliferation/Recruitment in Imiquimod-Induced Psoriasis Mouse Model. Eur J Immunol (2016) 46(6):1449–59. 10.1002/eji.201546070 27000947

[33] Mashiko S Bouguermouh S Rubio M Baba N Bissonnette R Sarfati M. Human Mast Cells are Major IL-22 Producers in Patients With Psoriasis and Atopic Dermatitis. J Allergy Clin Immunol (2015) 136(2):351–9.e1. 10.1016/j.jaci.2015.01.033 25792465

[34] Steinbach S Vordermeier HM Jones GJ. CD4+ and γδ T Cells are the Main Producers of IL-22 and IL-17A in Lymphocytes From Mycobacterium Bovis-Infected Cattle. Sci Rep (2016) 6:29990. 10.1038/srep29990 27427303 PMC4947955

[35] Lu Z Liu R Huang E Chu Y. MicroRNAs: New Regulators of IL-22. Cell Immunol (2016) 304-305:1–8. 10.1016/j.cellimm.2016.05.003 27221197

[36] Jia L Wu C. The Biology and Functions of Th22 Cells. Adv Exp Med Biol (2014) 841:209–30. 10.1007/978-94-017-9487-9_8 25261209

[37] Protopsaltis NJ Liang W Nudleman E Ferrara N. Interleukin-22 Promotes Tumor Angiogenesis. Angiogenesis (2019) 22(2):311–23. 10.1007/s10456-018-9658-x 30539314

[38] Andoh A Zhang Z Inatomi O Fujino S Deguchi Y Araki Y. Interleukin-22,a Member of the IL-10 Subfamily,Induces Inflammatory Responses in Colonic Subepithelial Myofibroblasts. Gastroenterology (2005) 129(3):969–84. 10.1053/j.gastro.2005.06.071 16143135

[39] Lejeune D Dumoutier L Constantinescu S Kruijer W Schuringa JJ Renauld JC. Interleukin-22 (IL-22) Activates the JAK/STAT and P38 MAP Kinase Pathways in a Rat Hepatoma Cell Line. Pathways that are shared with and distinct from IL-10. J Biol Chem (2002) 277(37):33676–82. 10.1074/jbc.M204204200 12087100

[40] Sonnenberg GF Fouser LA Artis D. Functional Biology of the IL-22-IL-22R Pathway in Regulating Immunity and Inflammation at Barrier Surfaces. Adv Immunol (2010) 107:1–29. 10.1016/B978-0-12-381300-8.00001-0 21034969

[41] Zhu J Zhou M Zhao X Mu M Cheng M. Blueberry,Combined With Probiotics,Alleviates non-Alcoholic Fatty Liver Disease *via* IL-22-mediated JAK1/STAT3/BAX signaling. Food Funct (2018) 9(12):6298–306. 10.1039/c8fo01227j 30411754

[42] He H Guttman-Yassky E. JAK Inhibitors for Atopic Dermatitis: An Update. Am J Clin Dermatol (2019) 20(2):181–92. 10.1007/s40257-018-0413-2 30536048

[43] Ciccia F Guggino G Rizzo A Bombardieri M Raimondo S Carubbi F. Interleukin (IL)-22 Receptor 1 is Over-Expressed in Primary Sjogren’s Syndrome and Sjogren-Associated non-Hodgkin Lymphomas and is Regulated by IL-18. Clin Exp Immunol (2015) 181(2):219–29. 10.1111/cei.12643 PMC4516437 25880879

[44] Che Y Su Z Xia L. Effects of IL-22 on Cardiovascular Diseases. Int Immunopharmacol (2020) 81:106277. 10.1016/j.intimp.2020.106277 32062077

[45] Pickert G Neufert C Leppkes M Zheng Y Wittkopf N Warntjen M. STAT3 Links IL-22 Signaling in Intestinal Epithelial Cells to Mucosal Wound Healing. J Exp Med (2009) 206(7):1465–72. 10.1084/jem.20082683 PMC2715097 19564350

[46] Nagalakshmi ML Rascle A Zurawski S Menon S de Waal Malefyt R. Interleukin-22 Activates STAT3 and Induces IL-10 by Colon Epithelial Cells. Int Immunopharmacol (2004) 4(5):679–91. 10.1016/j.intimp.2004.01.008 15120652

[47] Boniface K Bernard FX Garcia M Gurney AL Lecron JC Morel F. IL-22 Inhibits Epidermal Differentiation and Induces Proinflammatory Gene Expression and Migration of Human Keratinocytes. J Immunol (2005) 174(6):3695–702. 10.4049/jimmunol.174.6.3695 15749908

[48] Brand S Beigel F Olszak T Zitzmann K Eichhorst ST Otte JM. IL-22 is increased in active Crohn’s disease and promotes proinflammatory gene expression and intestinal epithelial cell migration. Am J Physiol Gastrointest Liver Physiol (2006) 290(4):G827–38. 10.1152/ajpgi.00513.2005 16537974

[49] Dumoutier L de Meester C Tavernier J Renauld JC. New Activation Modus of STAT3: A Tyrosine-Less Region of the Interleukin-22 Receptor Recruits STAT3 by Interacting With Its Coiled-Coil Domain. J Biol Chem (2009) 284(39):26377–84. 10.1074/jbc.M109.007955 PMC2785325 19632985

[50] Naher L Kiyoshima T Kobayashi I Wada H Nagata K Fujiwara H. STAT3 Signal Transduction Through Interleukin-22 in Oral Squamous Cell Carcinoma. Int J Oncol (2012) 41(5):1577–86. 10.3892/ijo.2012.1594 PMC3583669 22922995

[51] Wen H Liu Y Li J Wei D Liu D Zhao F. Inhibitory Effect and Mechanism of 1,25-Dihydroxy Vitamin D3 on RANKL Expression in Fibroblast-Like Synoviocytes and Osteoclast-Like Cell Formation Induced by IL-22 in Rheumatoid Arthritis. Clin Exp Rheumatol (2018) 36(5):798–805. 29465363

[52] Yeste A Mascanfroni ID Nadeau M Burns EJ Tukpah AM Santiago A. IL-21 Induces IL-22 Production in CD4+ T Cells. Nat Commun (2014) 5:3753. 10.1038/ncomms4753 24796415 PMC4157605

[53] Diaz-Zuniga J Melgar-Rodriguez S Monasterio G Pujol M Rojas L Alvarez C. Differential Human Th22-Lymphocyte Response Triggered by Aggregatibacter Actinomycetemcomitans Serotypes. Arch Oral Biol (2017) 78:26–33. 10.1016/j.archoralbio.2017.02.008 28189882

[54] Fujita H Nograles KE Kikuchi T Gonzalez J Carucci JA Krueger JG. Human Langerhans Cells Induce Distinct IL-22-Producing CD4+ T Cells Lacking IL-17 Production. Proc Natl Acad Sci United States America (2009) 106(51):21795–800. 10.1073/pnas.0911472106 PMC2799849 19996179

[55] Yoon J Leyva-Castillo JM Wang G Galand C Oyoshi MK Kumar L. IL-23 Induced in Keratinocytes by Endogenous TLR4 Ligands Polarizes Dendritic Cells to Drive IL-22 Responses to Skin Immunization. J Exp Med (2016) 213(10):2147–66. 10.1084/jem.20150376 PMC5032726 27551155

[56] Yang J Yang X Wang L Li M. B Cells Control Lupus Autoimmunity by Inhibiting Th17 and Promoting Th22 Cells. Cell Death Dis (2020) 11(3):164. 10.1038/s41419-020-2362-y 32127533 PMC7054432

[57] Fu D Song X Hu H Sun M Li Z Tian Z. Downregulation of RUNX3 Moderates the Frequency of Th17 and Th22 Cells in Patients With Psoriasis. Mol Med Rep (2016) 13(6):4606–12. 10.3892/mmr.2016.5108 PMC4878538 27082311

[58] Huang R Chen X Long Y Chen R. MiR-31 Promotes Th22 Differentiation Through Targeting Bach2 in Coronary Heart Disease. Biosci Rep (2019) 39(9):BSR20190986. 10.1042/BSR20190986 31501353 PMC6753318

[59] Piccinni MP Lombardelli L Logiodice F Kullolli O Maggi E Barkley MS. Medroxyprogesterone Acetate Decreases Th1,Th17,and Increases Th22 Responses *via* AHR Signaling Which Could Affect Susceptibility to Infections and Inflammatory Disease. Front Immunol (2019) 10:642. 10.3389/fimmu.2019.00642 31001262 PMC6456711

[60] Xiao C Xiao P Li X Li X Li H Chen Y. Cordyceps Sinensis may Inhibit Th22 Cell Chemotaxis to Improve Kidney Function in lgA Nephropathy. Am J Transl Res (2018) 10(3):857–65. PMC5883126 29636875

[61] Huang Y Xu T Li J. Th22 Cell is a Gradually Proved Potential Biomarker for Acute Coronary Syndrome. Mediators Inflammation (2014) 2014:813926. 10.1155/2014/813926 PMC4034660 24895489

[62] Huang YH Cao YF Jiang ZY Zhang S Gao F. Th22 Cell Accumulation is Associated With Colorectal Cancer Development. World J Gastroenterol (2015) 21(14):4216–24. 10.3748/wjg.v21.i14.4216 PMC4394082 25892871

[63] Cui D Zhong F Lin J Wu Y Long Q Yang X. Changes of Circulating Th22 Cells in Children With Hand, Foot, and Mouth Disease Caused by Enterovirus 71 Infection. Oncotarget (2017) 8(17):29370–82. 10.18632/oncotarget.14083 PMC5438737 28030850

[64] Zhang L Li JM Liu XG Ma DX Hu NW Li YG. Elevated Th22 Cells Correlated With Th17 Cells in Patients With Rheumatoid Arthritis. J Clin Immunol (2011) 31(4):606–14. 10.1007/s10875-011-9540-8 21556937

[65] Zhao L Jiang Z Jiang Y Ma N Zhang Y Feng L. IL-22+ CD4+ T Cells in Patients With Rheumatoid Arthritis. Int J Rheum Dis (2013) 16(5):518–26. 10.1111/1756-185x.12099 24164838

[66] Zhao L Jiang Z Jiang Y Ma N Wang K Zhang Y. IL-22+CD4+ T-Cells in Patients With Active Systemic Lupus Erythematosus. Exp Biol Med (Maywood) (2013) 238(2):193–9. 10.1177/1535370213477597 23576801

[67] Zhong W Jiang Y Ma H Wu J Jiang Z Zhao L. Elevated Levels of CCR6(+) T Helper 22 Cells Correlate With Skin and Renal Impairment in Systemic Lupus Erythematosus. Sci Rep (2017) 7(1):12962. 10.1038/s41598-017-13344-w 29021537 PMC5636893

[68] Yang X Weng Q Hu L Yang L Wang X Xiang X. Increased Interleukin-22 Levels in Lupus Nephritis and Its Associated With Disease Severity: A Study in Both Patients and Lupus-Like Mice Model. Clin Exp Rheumatol (2019) 37(3):400–7. 30299250

[69] Pan HF Zhao XF Yuan H Zhang WH Li XP Wang GH. Decreased Serum IL-22 Levels in Patients With Systemic Lupus Erythematosus. Clin Chim Acta (2009) 401(1-2):179–80. 10.1016/j.cca.2008.11.009 19046958

[70] Lin J Yue LH Chen WQ. Decreased Plasma IL-22 Levels and Correlations With IL-22-Producing T Helper Cells in Patients With New-Onset Systemic Lupus Erythematosus. Scand J Immunol (2014) 79(2):131–6. 10.1111/sji.12135 24313261

[71] Luk CC Tam LS Kwan BC Wong PC Ma TK Chow KM. Intrarenal and Urinary Th9 and Th22 Cytokine Gene Expression in Lupus Nephritis. J Rheumatol (2015) 42(7):1150–5. 10.3899/jrheum.140954 25979722

[72] Luan L Ding Y Han S Zhang Z Liu X. An Increased Proportion of Circulating Th22 and Tc22 Cells in Psoriasis. Cell Immunol (2014) 290(2):196–200. 10.1016/j.cellimm.2014.06.007 25046360

[73] Kagami S Rizzo HL Lee JJ Koguchi Y Blauvelt A. Circulating Th17,Th22,and Th1 Cells are Increased in Psoriasis. J Invest Dermatol (2010) 130(5):1373–83. 10.1038/jid.2009.399 PMC2892169 20032993

[74] Michalak-Stoma A Bartosińska J Kowal M Juszkiewicz-Borowiec M Gerkowicz A Chodorowska G. Serum Levels of Selected Th17 and Th22 Cytokines in Psoriatic Patients. Dis Markers (2013) 35(6):625–31. 10.1155/2013/856056 PMC3832981 24288431

[75] Xu W Li R Dai Y Wu A Wang H Cheng C. IL-22 Secreting CD4+ T Cells in the Patients With Neuromyelitis Optica and Multiple Sclerosis. J Neuroimmunol (2013) 261(1-2):87–91. 10.1016/j.jneuroim.2013.04.021 23726764

[76] Rolla S Bardina V De Mercanti S Quaglino P De Palma R Gned D. Th22 Cells are Expanded in Multiple Sclerosis and Are Resistant to IFN-β. J Leukocyte Biol (2014) 96(6):1155–64. 10.1189/jlb.5A0813-463RR 25097195

[77] Hu Y Li H Zhang L Shan B Xu X Li H. Elevated Profiles of Th22 Cells and Correlations With Th17 Cells in Patients With Immune Thrombocytopenia. Hum Immunol (2012) 73(6):629–35. 10.1016/j.humimm.2012.04.015 22537755

[78] Cao J Chen C Li L Ling-yu Z Zhen-yu L Zhi-ling Y. Effects of High-Dose Dexamethasone on Regulating Interleukin-22 Production and Correcting Th1 and Th22 Polarization in Immune Thrombocytopenia. J Clin Immunol (2012) 32(3):523–9. 10.1007/s10875-012-9649-4 22289995

[79] Zhan FX Li J Fang M Ding J Wang Q. Importance of Th22 Cell Disequilibrium in Immune Thrombocytopenic Purpura. Med Sci Monit (2018) 24:8767–72. 10.12659/msm.912528 PMC6289030 30510151

[80] Ruszkowski J Lisowska KA Pindel M Heleniak Z Dębska-Ślizień A. Witkowski JM. T Cells in IgA Nephropathy: Role in Pathogenesis, Clinical Significance and Potential Therapeutic Target. Clin Exp Nephrol (2019) 23(3):291–303. 10.1007/s10157-018-1665-0 30406499 PMC6394565

[81] Peng Z Tian J Cui X Xian W Sun H Li E. Increased Number of Th22 Cells and Correlation With Th17 Cells in Peripheral Blood of Patients With IgA Nephropathy. Hum Immunol (2013) 74(12):1586–91. 10.1016/j.humimm.2013.08.001 23978338

[82] Liang M Liwen Z Yun Z Yanbo D Jianping C. The Imbalance Between Foxp3(+)Tregs and Th1/Th17/Th22 Cells in Patients With Newly Diagnosed Autoimmune Hepatitis. J Immunol Res (2018) 2018:3753081. 10.1155/2018/3753081 30050955 PMC6040251

[83] Peng D Xu B Wang Y Guo H Jiang Y. A High Frequency of Circulating Th22 and Th17 Cells in Patients With New Onset Graves’ Disease. PloS One (2013) 8(7):e68446. 10.1371/journal.pone.0068446 23874630 PMC3708941

[84] Vitales-Noyola M Ramos-Levi AM Martínez-Hernández R Serrano-Somavilla A Sampedro-Nuñez M González-Amaro R. Pathogenic Th17 and Th22 Cells Are Increased in Patients With Autoimmune Thyroid Disorders. Endocrine (2017) 57(3):409–17. 10.1007/s12020-017-1361-y 28669056

[85] Bai X Sun J Wang W Shan Z Zheng H Li Y. Increased Differentiation of Th22 Cells in Hashimoto’s Thyroiditis. Endocr J (2014) 61(12):1181–90. 10.1507/endocrj.EJ14-0265 25242258

[86] Ruggeri RM Minciullo P Saitta S Giovinazzo S Certo R Campennì A. Serum Interleukin-22 (IL-22) Is Increased in the Early Stage of Hashimoto’s Thyroiditis Compared to Non-Autoimmune Thyroid Disease and Healthy Controls. Hormones (Athens) (2014) 13(3):338–44. 10.14310/horm.2002.1483 25079457

[87] Truchetet ME Brembilla NC Montanari E Allanore Y Chizzolini C. Increased Frequency of Circulating Th22 in Addition to Th17 and Th2 Lymphocytes in Systemic Sclerosis: Association With Interstitial Lung Disease. Arthrit Res Ther (2011) 13(5):R166. 10.1186/ar3486 PMC3308100 21996293

[88] Mathian A Parizot C Dorgham K Trad S Arnaud L Larsen M. Activated and Resting Regulatory T Cell Exhaustion Concurs With High Levels of Interleukin-22 Expression in Systemic Sclerosis Lesions. Ann Rheum Dis (2012) 71(7):1227–34. 10.1136/annrheumdis-2011-200709 22696687

[89] Brembilla NC Dufour AM Alvarez M Hugues S Montanari E Truchetet ME. IL-22 Capacitates Dermal Fibroblast Responses to TNF in Scleroderma. Ann Rheum Dis (2016) 75(9):1697–705. 10.1136/annrheumdis-2015-207477 26452537

[90] Toussirot É Laheurte C Gaugler B Gabriel D Saas P. Increased IL-22- and IL-17a-Producing Mucosal-Associated Invariant T Cells in the Peripheral Blood of Patients With Ankylosing Spondylitis. Front Immunol (2018) 9:1610. 10.3389/fimmu.2018.01610 30057583 PMC6053500

[91] Zerbini A Muratore F Boiardi L Ciccia F Bonacini M Belloni L. Increased Expression of Interleukin-22 in Patients With Giant Cell Arteritis. Rheumatol (Oxford) (2018) 57(1):64–72. 10.1093/rheumatology/kex334 28968695

[92] Lindahl H Guerreiro-Cacais AO Bedri SK Linnerbauer M Lindén M Abdelmagid N. IL-22 Binding Protein Promotes the Disease Process in Multiple Sclerosis. J Immunol (2019) 203(4):888–98. 10.4049/jimmunol.1900400 31292217

[93] Robat-Jazi B Hosseini M Shaygannejad V Nafissi S Rezaei A Mansourain M. High Frequency of Tc22 and Th22 Cells in Myasthenia Gravis Patients and Their Significant Reduction After Thymectomy. Neuroimmunomodulation (2018) 25(2):80–8. 10.1159/000490855 30071533

[94] Zheng S Dou C Xin N Wang J Wang J Li P. Expression of Interleukin-22 in Myasthenia Gravis. Scand J Immunol (2013) 78(1):98–107. 10.1111/sji.12057 23617779

[95] Li S Yin H Zhang K Wang T Yang Y Liu X. Effector T Helper Cell Populations Are Elevated in the Bone Marrow of Rheumatoid Arthritis Patients and Correlate With Disease Severity. Sci Rep (2017) 7(1):4776. 10.1038/s41598-017-05014-8 28684770 PMC5500482

[96] Zhong W Zhao L Liu T Jiang Z. IL-22-Producing CD4+T Cells in the Treatment Response of Rheumatoid Arthritis to Combination Therapy With Methotrexate and Leflunomide. Sci Rep (2017) 7:41143. 10.1038/srep41143 28117352 PMC5259708

[97] van Hamburg JP Corneth OB Paulissen SM Davelaar N Asmawidjaja PS Mus AM. IL-17/Th17 Mediated Synovial Inflammation is IL-22 Independent. Ann Rheum Dis (2013) 72(10):1700–7. 10.1136/annrheumdis-2012-202373 23328939

[98] Miyazaki Y Nakayamada S Kubo S Nakano K Iwata S Miyagawa I. Th22 Cells Promote Osteoclast Differentiation *via* Production of IL-22 in Rheumatoid Arthritis. Front Immunol (2018) 9:2901. 10.3389/fimmu.2018.02901 30619268 PMC6295478

[99] Bakheet SA Ansari MA Nadeem A Attia SM Alhoshani AR Gul G. CXCR3 Antagonist AMG487 Suppresses Rheumatoid Arthritis Pathogenesis and Progression by Shifting the Th17/Treg Cell Balance. Cell Signal (2019) 64:109395. 10.1016/j.cellsig.2019.109395 31449849

[100] Zhao L Ma H Jiang Z Jiang Y Ma N. Immunoregulation Therapy Changes the Frequency of Interleukin (IL)-22+ CD4+ T Cells in Systemic Lupus Erythematosus Patients. Clin Exp Immunol (2014) 177(1):212–8. 10.1111/cei.12330 PMC4089170 24635166

[101] Rekik R Smiti Khanfir M Larbi T Zamali I Beldi-Ferchiou A Kammoun O. Impaired TGF-β Signaling in Patients With Active Systemic Lupus Erythematosus is Associated With an Overexpression of IL-22. Cytokine (2018) 108:182–9. 10.1016/j.cyto.2018.04.011 29684755

[102] Yang XY Wang HY Zhao XY Wang LJ Lv QH Wang QQ. Th22, But Not Th17 Might be a Good Index to Predict the Tissue Involvement of Systemic Lupus Erythematosus. J Clin Immunol (2013) 33(4):767–74. 10.1007/s10875-013-9878-1 23435610

[103] Wang R Zeng YL Qin HM Lu YL Huang HT Lei M. Association of Interleukin 22 Gene Polymorphisms and Serum IL-22 Level With Risk of Systemic Lupus Erythematosus in a Chinese Population. Clin Exp Immunol (2018) 193(2):143–51. 10.1111/cei.13133 PMC6046499 29603203

[104] Farber DL Yudanin NA Restifo NP. Human Memory T Cells: Generation,Compartmentalization and Homeostasis. Nat Rev Immunol (2014) 14(1):24–35. 10.1038/nri3567 24336101 PMC4032067

[105] Owczarczyk Saczonek A Krajewska-Włodarczyk M Kasprowicz-Furmańczyk M Placek W. Immunological Memory of Psoriatic Lesions. Int J Mol Sci (2020) 21(2):625. 10.3390/ijms21020625 PMC7014148 31963581

[106] Guilloteau K Paris I Pedretti N Boniface K Juchaux F Huguier V. Skin Inflammation Induced by the Synergistic Action of IL-17a,IL-22 IL-1{Alpha},and TNF-{Alpha} Recapitulates Some Features of Psoriasis. J Immunol (2010) 184(9):5263–70. 10.4049/jimmunol.0902464 20335534

[107] Guttman-Yassky E Nograles KE Krueger JG. Contrasting Pathogenesis of Atopic Dermatitis and Psoriasis–Part II: Immune Cell Subsets and Therapeutic Concepts. J Allergy Clin Immunol (2011) 127(6):1420–32. 10.1016/j.jaci.2011.01.054 21419481

[108] Yang X Zheng SG. Interleukin-22: A Likely Target for Treatment of Autoimmune Diseases. Autoimmun Rev (2014) 13(6):615–20. 10.1016/j.autrev.2013.11.008 PMC3966954 24418299

[109] Georgescu SR Tampa M Caruntu C Sarbu MI Mitran CI Mitran MI. Advances in Understanding the Immunological Pathways in Psoriasis. Int J Mol Sci (2019) 20(3):739. 10.3390/ijms20030739 PMC6387410 30744173

[110] Zhang N Pan HF Ye DQ. Th22 in Inflammatory and Autoimmune Disease: Prospects for Therapeutic Intervention. Mol Cell Biochem (2011) 353(1-2):41–6. 10.1007/s11010-011-0772-y 21384158

[111] Van Belle AB de Heusch M Lemaire MM Hendrickx E Warnier G Dunussi-Joannopoulos K. IL-22 is Required for Imiquimod-Induced Psoriasiform Skin Inflammation in Mice. J Immunol (2012) 188(1):462–9. 10.4049/jimmunol.1102224 22131335

[112] Diani M Altomare G. Reali E. T Helper Cell Subsets in Clinical Manifestations of Psoriasis. J Immunol Res (2016) 2016:7692024. 10.1155/2016/7692024 27595115 PMC4995329

[113] Cheuk S Wikén M Blomqvist L Nylén S Talme T Ståhle M. Epidermal Th22 and Tc17 Cells Form a Localized Disease Memory in Clinically Healed Psoriasis. J Immunol (2014) 192(7):3111–20. 10.4049/jimmunol.1302313 PMC3962894 24610014

[114] Le ST Merleev AA Luxardi G Shimoda M Adamopoulos IE Tsoi LC. 2d Visualization of the Psoriasis Transcriptome Fails to Support the Existence of Dual-Secreting IL-17a/IL-22 Th17 T Cells. Front Immunol (2019) 10:589. 10.3389/fimmu.2019.00589 31019502 PMC6458264

[115] Almolda B Costa M Montoya M González B Castellano B. Increase in Th17 and T-Reg Lymphocytes and Decrease of IL22 Correlate With the Recovery Phase of Acute EAE in Rat. PloS One (2011) 6(11):e27473. 10.1371/journal.pone.0027473 22110656 PMC3217052

[116] Muls N Nasr Z Dang HA Sindic C van Pesch V. IL-22,GM-CSF and IL-17 in Peripheral CD4+ T Cell Subpopulations During Multiple Sclerosis Relapses and Remission. Impact of Corticosteroid Therapy. PloS One (2017) 12(3):e0173780. 10.1371/journal.pone.0173780 28301515 PMC5354390

[117] Wing AC Hygino J Ferreira TB Kasahara TM Barros PO Sacramento PM. Interleukin-17- and Interleukin-22-Secreting Myelin-Specific CD4(+) T Cells Resistant to Corticoids are Related With Active Brain Lesions in Multiple Sclerosis Patients. Immunology (2016) 147(2):212–20. 10.1111/imm.12552 PMC4717237 26781085

[118] Alvarenga-Filho H Salles M Hygino J Ferreira TB Sacramento PM Monteiro C. Fatigue Favors *in vitro* Th1 and Th17-like cell expansion and reduces corticoid sensitivity in MS patients. J Neuroimmunol (2017) 303:81–9. 10.1016/j.jneuroim.2016.12.013 28065580

[119] Perriard G Mathias A Enz L Canales M Schluep M Gentner M. Interleukin-22 is Increased in Multiple Sclerosis Patients and Targets Astrocytes. J Neuroinflamm (2015) 12:119. 10.1186/s12974-015-0335-3 PMC4480507 26077779

[120] Bai Z Chen D Wang L Zhao Y Liu T Yu Y. Cerebrospinal Fluid and Blood Cytokines as Biomarkers for Multiple Sclerosis: A Systematic Review and Meta-Analysis of 226 Studies With 13,526 Multiple Sclerosis Patients. Front Neurosci (2019) 13:1026. 10.3389/fnins.2019.01026 31636528 PMC6787166

[121] Magliozzi R Howell OW Nicholas R Cruciani C Castellaro M Romualdi C. Inflammatory Intrathecal Profiles and Cortical Damage in Multiple Sclerosis. Ann Neurol (2018) 83(4):739–55. 10.1002/ana.25197 29518260

[122] Jaime-Pérez JC Turrubiates-Hernández GA López-Silva LJ Salazar-Riojas R Gómez-Almaguer D. Early Changes in IL-21, IL-22, CCL2, and CCL4 Serum Cytokines After Outpatient Autologous Transplantation for Multiple Sclerosis: A Proof of Concept Study. Clin Transplant (2020) 34(12):e14114. 10.1111/ctr.14114 33048389

[123] Zhen J Yuan J Fu Y Zhu R Wang M Chang H. IL-22 Promotes Fas Expression in Oligodendrocytes and Inhibits FOXP3 Expression in T Cells by Activating the NF-κb Pathway in Multiple Sclerosis. Mol Immunol (2017) 82:84–93. 10.1016/j.molimm.2016.12.020 28038358

[124] Beyeen AD Adzemovic MZ Ockinger J Stridh P Becanovic K Laaksonen H. IL-22RA2 Associates With Multiple Sclerosis and Macrophage Effector Mechanisms in Experimental Neuroinflammation. J Immunol (2010) 185(11):6883–90. 10.4049/jimmunol.1001392 21041731

[125] Shabgah AG Navashenaq JG Shabgah OG Mohammadi H Sahebkar A. Interleukin-22 in Human Inflammatory Diseases and Viral Infections. Autoimmun Rev (2017) 16(12):1209–18. 10.1016/j.autrev.2017.10.004 29037907

[126] Laaksonen H Guerreiro-Cacais AO Adzemovic MZ Parsa R Zeitelhofer M Jagodic M. The Multiple Sclerosis Risk Gene IL22RA2 Contributes to a More Severe Murine Autoimmune Neuroinflammation. Genes Immun (2014) 15(7):457–65. 10.1038/gene.2014.36 25008863

[127] Kostic M Zivkovic N Cvetanovic A Marjanović G. CD4(+) T Cell Phenotypes in the Pathogenesis of Immune Thrombocytopenia. Cell Immunol (2020) 351:104096. 10.1016/j.cellimm.2020.104096 32199587

[128] Cao J Chen C Zeng L Li L Li X Li Z. Elevated Plasma IL-22 Levels Correlated With Th1 and Th22 Cells in Patients With Immune Thrombocytopenia. Clin Immunol (2011) 141(1):121–3. 10.1016/j.clim.2011.05.003 21652269

[129] Jernås M Hou Y Strömberg Célind F Shao L Wang Q Ju X. Altered Cytokine Levels in Pediatric ITP. Platelets (2015) 26(6):589–92. 10.3109/09537104.2014.974526 25806433

[130] Wang Q Li J Yu TS Liu Y Li K Liu S. Disrupted Balance of CD4(+) T-Cell Subsets in Bone Marrow of Patients With Primary Immune Thrombocytopenia. Int J Biol Sci (2019) 15(13):2798–814. 10.7150/ijbs.33779 PMC6909963 31853219

[131] Lai KN Tang SC Schena FP Novak J Tomino Y Fogo AB. IgA Nephropathy. Nat Rev Dis Primers (2016) 2:16001. 10.1038/nrdp.2016.1 27189177

[132] Gan L Zhu M Li X Chen C Meng T Pu J. Tonsillitis Exacerbates Renal Injury in IgA Nephropathy Through Promoting Th22 Cells Chemotaxis. Int Urol Nephrol (2018) 50(7):1285–92. 10.1007/s11255-018-1792-2 29549623

[133] Xiao C Xiao P Li X Huang G Li H Chen Y. Streptococcus may Aggravate Inflammatory Damage in Chronic Nephritis *via* the chemotaxis of Th22 cells. Am J Transl Res (2019) 11(12):7432–40. PMC6943466 31934290

[134] Weathington NM Snavely CA Chen BB Zhao J Zhao Y Mallampalli RK. Glycogen Synthase Kinase-3β Stabilizes the Interleukin (IL)-22 Receptor From Proteasomal Degradation in Murine Lung Epithelia. J Biol Chem (2014) 289(25):17610–9. 10.1074/jbc.M114.551747 PMC4067196 24742671

[135] Gan L Zhou Q Li X Chen C Meng T Pu J. Intrinsic Renal Cells Induce Lymphocytosis of Th22 Cells From IgA Nephropathy Patients Through B7-CTLA-4 and CCL-CCR Pathways. Mol Cell Biochem (2018) 441(1-2):191–9. 10.1007/s11010-017-3185-8 28875388

[136] Xiao C Zhou Q Li X Li H Zhong Y Meng T. Losartan and Dexamethasone may Inhibit Chemotaxis to Reduce the Infiltration of Th22 Cells in IgA Nephropathy. Int Immunopharmacol (2017) 42:203–8. 10.1016/j.intimp.2016.11.025 27930971

[137] Gan L Li X Zhu M Chen C Luo H Zhou Q. Acteoside Relieves Mesangial Cell Injury by Regulating Th22 Cell Chemotaxis and Proliferation in IgA Nephropathy. Ren Fail (2018) 40(1):364–70. 10.1080/0886022x.2018.1450762 PMC6014492 29708439

[138] Suh JS Cho SH Chung JH Moon A Park YK. Cho BS. A Polymorphism of Interleukin-22 Receptor Alpha-1 Is Associated With the Development of Childhood IgA Nephropathy. J Interferon Cytokine Res (2013) 33(10):571–7. 10.1089/jir.2012.0097 PMC3793651 23659670

[139] Seki E Brenner DA. Recent Advancement of Molecular Mechanisms of Liver Fibrosis. J Hepatobiliary Pancreat Sci (2015) 22(7):512–8. 10.1002/jhbp.245 PMC4668270 25869468

[140] Radaeva S Sun R Pan HN Hong F Gao B. Interleukin 22 (IL-22) Plays a Protective Role in T Cell-Mediated Murine Hepatitis: IL-22 Is a Survival Factor for Hepatocytes *via* STAT3 activation. Hepatology (2004) 39(5):1332–42. 10.1002/hep.20184 15122762

[141] Feng D. Interleukin-22, Liver Progenitor Cells,and Liver Cancer. Hepatology (2014) 60(1):427–8. 10.1002/hep.26899 24306993

[142] Lai R Xiang X Mo R Bao R Wang P Guo S. Protective Effect of Th22 Cells and Intrahepatic IL-22 in Drug Induced Hepatocellular Injury. J Hepatol (2015) 63(1):148–55. 10.1016/j.jhep.2015.02.004 25681556

[143] Mo R Wang P Lai R Li F Liu Y Jiang S. Persistently Elevated Circulating Th22 Reversely Correlates With Prognosis in HBV-Related Acute-on-Chronic Liver Failure. J Gastroenterol Hepatol (2017) 32(3):677–86. 10.1111/jgh.13537 27548078

[144] Thomas-Dupont P Remes-Troche JM Izaguirre-Hernández IY Sánchez-Vargas LA Maldonado-Rentería Mde J Hernández-Flores KG. Elevated circulating levels of IL-21 and IL-22 define a cytokine signature profile in type 2 autoimmune hepatitis patients. Ann Hepatol (2016) 15(4):550–8. 27236154

[145] Li Q Wang B Mu K Zhang JA. The Pathogenesis of Thyroid Autoimmune Diseases: New T Lymphocytes - Cytokines Circuits Beyond the Th1-Th2 Paradigm. J Cell Physiol (2019) 234(3):2204–16. 10.1002/jcp.27180 30246383

[146] Song RH Yu ZY Qin Q Wang X Muhali FS Shi LF. Different Levels of Circulating Th22 Cell and its Related Molecules in Graves’ Disease and Hashimoto’s Thyroiditis. Int J Clin Exp Pathol (2014) 7(7):4024–31. PMC4129015 25120780

[147] Binks S Vincent A Palace J. Myasthenia Gravis: A Clinical-Immunological Update. J Neurol (2016) 263(4):826–34. 10.1007/s00415-015-7963-5 PMC4826656 26705120

[148] Wang L Zhang Y Zhu M Feng J Han J Zhu J. Effects of Follicular Helper T Cells and Inflammatory Cytokines on Myasthenia Gravis. Curr Mol Med (2019) 19(10):739–45. 10.2174/1566524019666190827162615 31453784

[149] Liu M Wu W Sun X Yang J Xu J Fu W. New Insights Into CD4(+) T Cell Abnormalities in Systemic Sclerosis. Cytokine Growth Factor Rev (2016) 28:31–6. 10.1016/j.cytogfr.2015.12.002 26724976

[150] Zhang M. Zhang S. T Cells in Fibrosis and Fibrotic Diseases. Front Immunol (2020) 11:1142. 10.3389/fimmu.2020.01142 32676074 PMC7333347

[151] Furuzawa-Carballeda J Sánchez-Guerrero J Betanzos JL Enriquez AB Avila-Casado C Llorente L. Differential Cytokine Expression and Regulatory Cells in Patients With Primary and Secondary Sjögren’s Syndrome. Scand J Immunol (2014) 80(6):432–40. 10.1111/sji.12224 25346207

[152] Ciccia F Guggino G Rizzo A Ferrante A Raimondo S Giardina A. Potential Involvement of IL-22 and IL-22-Producing Cells in the Inflamed Salivary Glands of Patients With Sjogren’s Syndrome. Ann Rheum Dis (2012) 71(2):295–301. 10.1136/ard.2011.154013 21979002

[153] Ciccia F Guggino G Giardina A Ferrante A Carrubbi F Giacomelli R. The Role of Innate and Lymphoid IL-22-Producing Cells in the Immunopathology of Primary Sjögren’s Syndrome. Expert Rev Clin Immunol (2014) 10(4):533–41. 10.1586/1744666x.2014.884461 24490899

[154] Shen H Goodall JC Hill Gaston JS. Frequency and Phenotype of Peripheral Blood Th17 Cells in Ankylosing Spondylitis and Rheumatoid Arthritis. Arthritis Rheum (2009) 60(6):1647–56. 10.1002/art.24568 19479869

[155] Uppal SK Kearns DG Chat VS Han G Wu JJ. Review and Analysis of Biologic Therapies Currently in Phase II and Phase III Clinical Trials for Atopic Dermatitis. J Dermatolog Treat (2020) 1–11. 10.1080/09546634.2020.1775775 32507066

[156] Guttman-Yassky E Brunner PM Neumann AU Khattri S Pavel AB Malik K. Efficacy and Safety of Fezakinumab (an IL-22 Monoclonal Antibody) in Adults With Moderate-to-Severe Atopic Dermatitis Inadequately Controlled by Conventional Treatments: A Randomized, Double-Blind, Phase 2a Trial. J Am Acad Dermatol (2018) 78(5):872–81.e6. 10.1016/j.jaad.2018.01.016 29353025 PMC8711034

[157] Brunner PM Pavel AB Khattri S Leonard A Malik K Rose S. Baseline IL-22 Expression in Patients With Atopic Dermatitis Stratifies Tissue Responses to Fezakinumab. J Allergy Clin Immunol (2019) 143(1):142–54. 10.1016/j.jaci.2018.07.028 30121291

[158] Perusina Lanfranca M Lin Y Fang J Zou W Frankel T. Biological and Pathological Activities of Interleukin-22. J Mol Med (Berl) (2016) 94(5):523–34. 10.1007/s00109-016-1391-6 PMC4860114 26923718

